# Oral pain and comorbidities in an edentulous older population: A k-prototypes cluster analysis

**DOI:** 10.1371/journal.pone.0319819

**Published:** 2025-03-13

**Authors:** Nontawat Chuinsiri, Natthapol Thinsathid

**Affiliations:** 1 Institute of Dentistry, Suranaree University of Technology, Nakhon Ratchasima, Thailand; 2 Oral Health Center, Suranaree University of Technology Hospital, Suranaree University of Technology, Nakhon Ratchasima, Thailand; Far Eastern Memorial Hospital, TAIWAN

## Abstract

Non-odontogenic oral pain is prevalent among the older people and significantly impacts their quality of life. Non-odontogenic oral pain is usually persistent and accompanied by comorbidities such as psychosocial distress and sleep-related problems, which further complicate pain management. The relationship between non-odontogenic oral pain and comorbidities in the older people, however, has not been well documented. This study aimed to identify the factors associated with non-odontogenic oral pain in an edentulous older population and to subgroup this population based on the patterns of oral pain and its associated factors. In this cross-sectional study, data from completely edentulous individuals in the National Health and Nutrition Examination Survey for the period from 2017 to 2020 March (pre-pandemic) were analysed. Associations and correlations between oral pain and 46 other variables, including demographic, questionnaire, examination and laboratory data, were investigated using Pearson’s chi-squared test and Spearman’s rank correlation test. A *p* value of < 0.05 was considered statistically significant. Clustering of the data was performed using the k-prototypes algorithm, an unsupervised machine learning. Approximately 42% of the edentulous older people experienced oral pain. ‘Having been told to take daily low-dose aspirin’ was significantly associated with oral pain. Oral pain was positively correlated with depressive symptoms and excessive daytime sleepiness (EDS), and negatively correlated with diastolic blood pressure, red blood cell count, haemoglobin level and haematocrit. The k-prototypes algorithm identified a cluster characterised by frequent oral pain, depression and EDS. This study identified distinct patterns of comorbidities among edentulous older people living with oral pain.

## Introduction

Non-odontogenic orofacial pain often poses as a chronic problem that significantly impacts the quality of life for many individuals [1]. Older age has been reported as one risk factor for chronic pain [2], contributing to the high prevalence of orofacial pain among the older people. Studies indicate that approximately 17% of older people experience orofacial pain within a given year [3], with the prevalence rising to 26% among those with cognitive impairments [4]. Common types of non-odontogenic orofacial pain in the older people include burning mouth syndrome (BMS), temporomandibular disorders (TMDs) and trigeminal neuralgia [3,5,6]. This type of pain is often multifactorial in nature and usually presents with several risk factors and comorbidities, including emotional instability and systemic diseases [6,7]. Despite this, comorbidities of non-odontogenic orofacial pain in the older people remains understudied. Considering that the global population is moving towards the superaged society [8], it is crucial to also investigate these factors and comorbidities of non-odontogenic orofacial pain in the older people.

The biopsychosocial model of pain states that biological, psychological and social factors influence the experience of pain; thus, addressing each of these factors is essential for effective pain management [9]. A meta-analysis reveals that multimorbidity is more prevalent in individuals aged 59 and above [10]. The prevalence of multimorbidity increases significantly with age, with hypertension, depression/anxiety and chronic pain being the most commonly observed conditions [11]. With the increasing tendency of non-odontogenic pain-related comorbidities such as depression [12] and insomnia [13] in the older people, their existing pain conditions can become even more severe and difficult to manage, requiring special attention. Given the vulnerability of older people to multimorbidity, identifying factors associated with non-odontogenic pain can provide valuable insights for a more holistic approach to pain management.

Unsupervised machine learning is a clustering technique to uncover patterns and relationships within datasets, such as identifying disease subtypes and associated factors, without prior labelling [14]. K-prototypes cluster analysis is a hybrid clustering method that combines k-means and k-modes algorithms, effectively handling datasets with both numerical and categorical variables [15]. This makes it particularly valuable in clinical research, where data often include continuous measures and categorical variables. The k-prototypes algorithm is well-validated and has been widely used in medical studies, such as those involving respiratory diseases, cardiovascular diseases and diabetes, where it identifies patient subgroups based on multiple characteristics [16–18]. A previous benchmark analysis conducted on a dataset of patients with multimorbidity demonstrated that the k-prototypes algorithm outperformed other distance-based methods and performed similarly to the Latent Class Model method in identifying clusters with distinct characteristics [19]. Thus, how the edentulous older population could be clustered based on the patterns of oral pain and its associated factors was the question of this investigation.

This study aimed to identify the factors associated with non-odontogenic oral pain in an edentulous older population and to subgroup this population based on the patterns of oral pain and its associated factors using the k-prototypes cluster analysis.

## Materials and methods

This cross-sectional study complies with Strengthening the Reporting of Observational Studies in Epidemiology Guidelines [20].

### Dataset and study design

This study utilised the publicly available anonymised dataset collected from 15,560 non-institutionalised civilian residents of the USA between 2017 and 2020 March (pre-pandemic) as part of the National Health and Nutrition Examination Survey (NHANES) [21]. Hence, approval from an institutional review board was not required.

**Fig 1:**
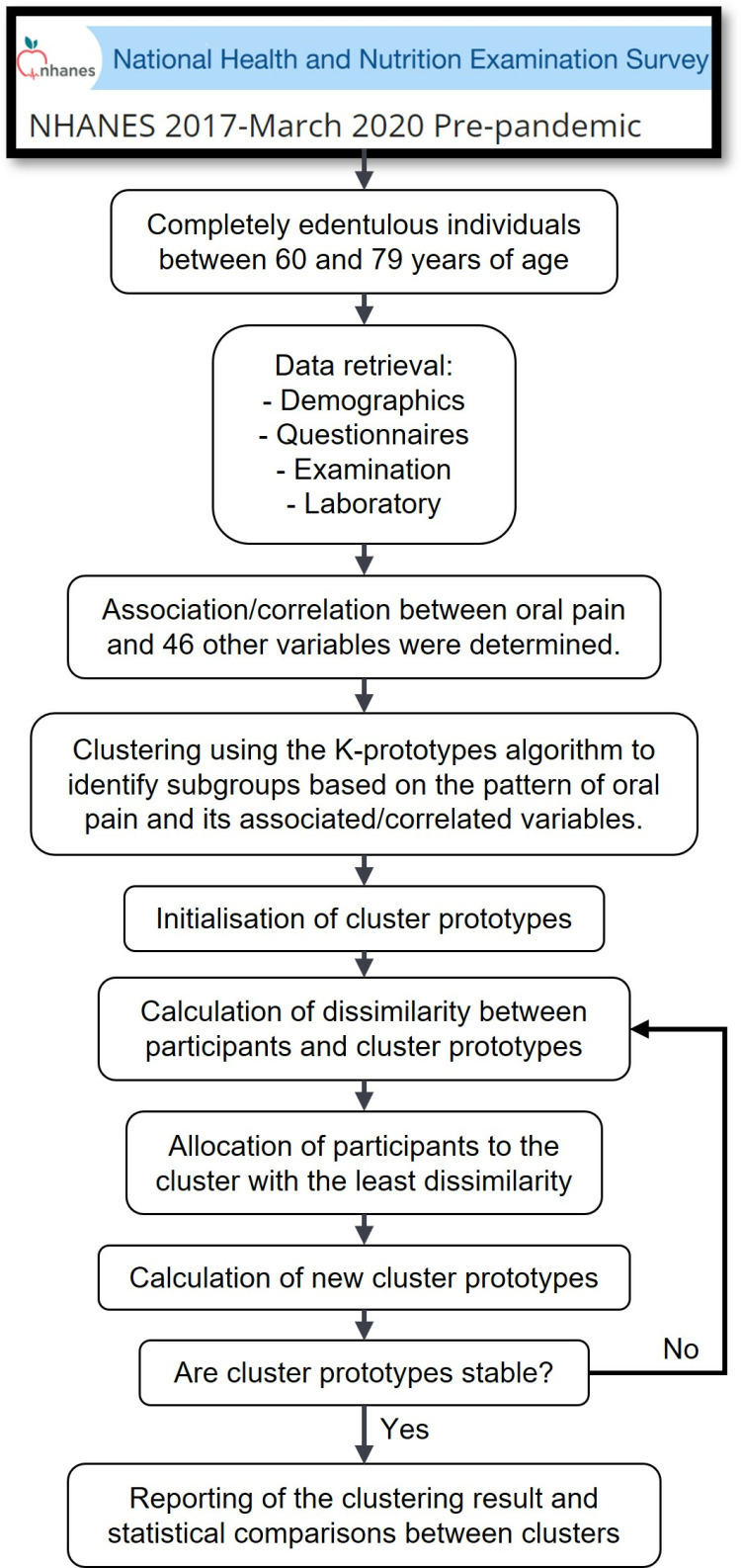
Schematic depicting the flow of the study.

An overview of the study design is shown in Fig 1. We retrieved participants’ data including demographic, questionnaire, examination and laboratory data (S1 Table). To address the research question, completely edentulous participants, who were between 60 and 79 years of age and provided data regarding oral pain, were first identified. In the NHANES dataset, individuals aged 80 and above are topcoded at 80 years, and these individuals were excluded from the analyses. Oral pain was assessed by asking, ‘How often during the last year have you had painful aching anywhere in your mouth?’. Responses were categorised as: 1) never, 2) hardly ever, 3) occasionally to fairly often and 4) very often. The assessment of other variables and possible responses can be found in S1 Table. The association/correlation between oral pain and other variables (a total of 46) was determined using appropriate statistical tests as described below. Cluster analysis was then performed to identify subgroups based on the pattern of oral pain and its significantly associated/correlated variables identified in the preceding steps. Statistical comparisons between clusters were performed as outlined below.

### Determination of association/correlation between oral pain and other variables

Python version 3.8 was used for statistical analyses. The association between oral pain and categorical variables was investigated using Pearson’s chi-squared test, while Spearman’s rank correlation test was used to investigate correlations between oral pain and numerical variables. Variables with more than 25% missing data were excluded from the analyses. A *p* value of < 0.05 was considered statistically significant.

### Clustering method

Data pre-processing and clustering were performed using Python version 3.8. Numerical variables were transformed to be within the range of 0 and 1 using the minimum-maximum scaling method from the Scikit-learn module (https://scikit-learn.org/stable/modules/preprocessing.html). This method rescales the data by subtracting the minimum value of each variable and then dividing by the range (the difference between the maximum and minimum values).

Clustering was performed using the k-prototypes algorithm, which combines the principles of k-means (for numerical data) and k-modes (for categorical data). The algorithm uses a combination of Euclidean distance for numerical features and matching dissimilarity measures for categorical features to determine cluster assignments. [15,19]. We used the open-source k-prototypes algorithm from the kmodes module (https://pypi.org/project/kmodes/).

The process began by initialising prototypes (representative collections of variables) for each cluster. Each participant was then allocated to the cluster whose prototype was most similar, based on dissimilarity, which is a measure of how different two objects are from each other. For numerical variables, dissimilarity was calculated using Euclidean distance, based on the Pythagorean theorem, which measures the straight-line distance between data points in a multi-dimensional space. For categorical variables, dissimilarity was measured using Hamming distance, which counts the number of mismatched categorical attributes between a participant and the cluster prototype. The total dissimilarity was computed as the sum of these two components, and participants were allocated to the cluster with the least dissimilarity. The algorithm iteratively refined the clusters by updating their prototypes and reallocating participants based on the recalculated dissimilarities. This process continued until a stable clustering solution was reached, meaning no participant had moved to a different cluster [15,22].

The standard elbow method was employed to determine the optimum number of clusters (k) [23]. The k-prototypes algorithm was iterated on different values of k from 1 to 15. Dissimilarity was calculated and plotted against respective k values. The value of k at which the dissimilarity started to decrease linearly was selected as the optimum number of clusters.

### Cluster comparisons

After determining the optimal number of clusters, participants were assigned to the appropriate clusters based on their dissimilarity to the cluster prototype, as described above. Normality of the variables was assessed using the Shapiro-Wilk test. Differences in numerical variables across clusters were analysed with the Kruskal-Wallis test, followed by Dunn’s multiple comparisons test. Pearson’s chi-squared test was used to assess the distribution of categorical variables between clusters. Statistical differences between clusters were conducted using GraphPad Prism version 10.4 (GraphPad Software, Boston, MA, USA). A *p* value of < 0.05 was considered statistically significant.

## Results

### Sociodemographic characteristics of the included population

Of the 13,772 participants who underwent a dentition examination, a total of 333 edentulous participants, who were aged 60 to 79 years (mean = 68.8; standard deviation = 5.6) and also provided data about oral pain frequency, were identified. Fifty-five percent (183/333) of the included participants were male. The highest level of education obtained by the participants was as follow: less than 9^th^ grade (n = 54), 9-11^th^ grade (n = 61), high school graduate/GED or equivalent (n = 105), some college or AA degree (n = 93) and college graduate or above (n = 20). The mean family income-to-poverty ratio was 1.86 (standard deviation = 1.24). One hundred and ninety-four participants (58.3%) never had oral pain. Ninety participants (27%) hardly ever had oral pain. Forty-one participants (12.3%) occasionally to fairly often had oral pain. Eight participants (2.4%) very often had oral pain.

### Association and correlation between variables

**Fig 2:**
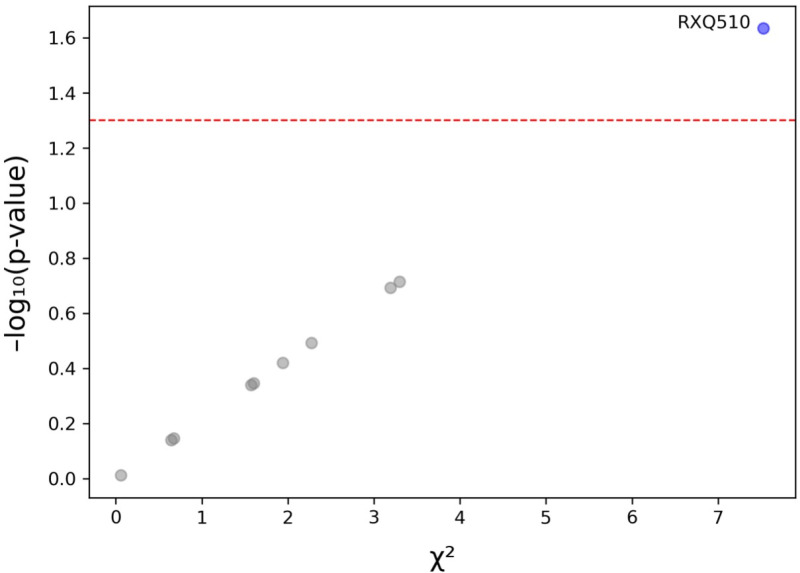
Association between oral pain and categorical variables. Chi-squared values and p-values (on a negative logarithmic scale) for each categorical variables in relation to oral pain are plotted. The horizontal dotted red line represents a *p* value of 0.05. Only ‘having been told to take daily low-dose aspirin’ (RXQ510) passed this threshold and was significantly associated with oral pain.

The Pearson’s chi-squared test showed that ‘having been told by doctors or other health care providers to take daily low-dose aspirin’ was significantly associated with oral pain (*p* = 0.023, n = 333, Fig 2). A slightly higher percentage of participants who were told to take daily low-dose aspirin reported ‘occasionally to fairly often’ and ‘very often’ oral pain (15.5% vs 13%, Table 1).

**Table 1:** A contingency table shows the frequency distribution of the included participants according to their oral pain reports and whether they have been told to take daily low-dose aspirin.

Oral pain report				
Having been told to take daily low-dose aspirin	Never (n = 194)	Hardly ever (n = 90)	Occasionally to fairly often (n = 41)	Very often (n = 8)
No (n = 131)	n = 88 (67%)	n = 26 (20%)	n = 14 (11%)	n = 3 (2%)
Yes (n = 202)	n = 106 (52.5%)	n = 64 (32%)	n = 27 (13%)	n = 5 (2.5%)

Data are presented as the number of participants with the percentage of row total shown in the parentheses.

**Fig 3:**
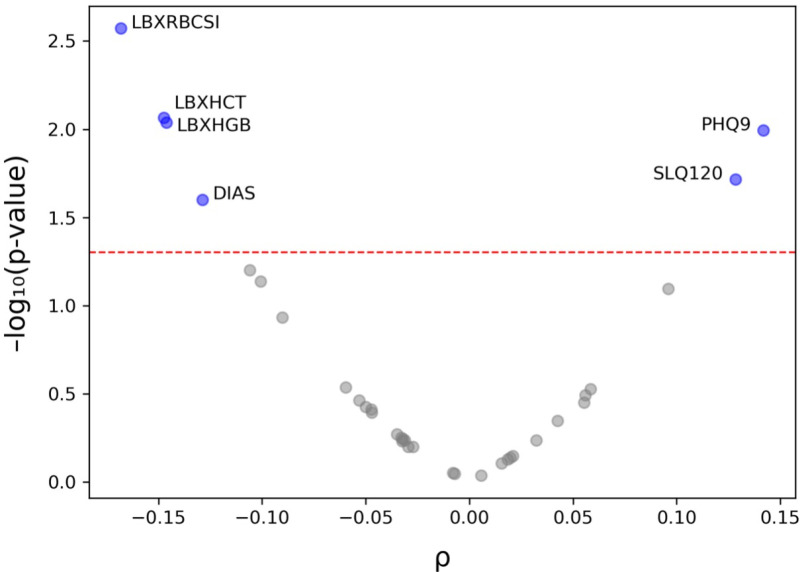
Association between oral pain and numerical variables. Spearman’s rho values and p-values (on a negative logarithmic scale) for each numerical variables in relation to oral pain are plotted. The horizontal dotted red line represents a *p* value of 0.05. Depression (PHQ9) and daytime sleepiness (SLQ120) were significantly positively correlated with oral pain. Red blood cell count (LBXRBCSI), haemoglobin level (LBXHGB), haematocrit (LBXHCT) and diastolic blood pressure (DIAS) were significantly negatively correlated with oral pain.

The Spearman’s rank correlation test showed that oral pain was positively correlated with depressive symptoms (*p* = 0.01, n = 328), as assessed by the Patient Health Questionnaire-9 (PHQ9), and with excessive daytime sleepiness (EDS, *p* = 0.02, n = 332), assessed by asking the participants, ‘how often they felt excessively or overly sleepy during the day’. Average diastolic blood pressure (DBP) from three measurements was found to be negatively correlated with oral pain (*p* = 0.025, n = 303). Oral pain was also negatively correlated with three laboratory-based variables: red blood cell (RBC) count (*p* = 0.003, n = 317), haemoglobin level (*p* = 0.01, n = 317) and haematocrit (*p* = 0.01, n = 317) (Fig 3).

### Clustering

**Fig 4:**
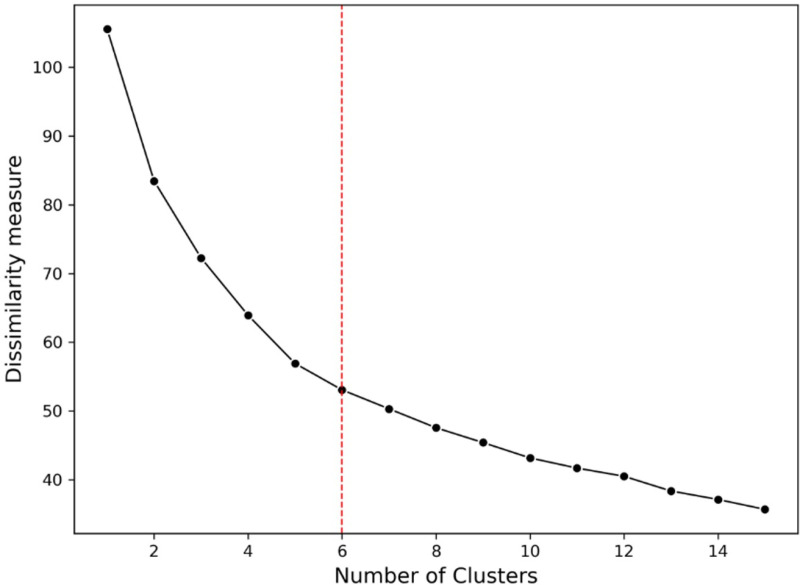
Elbow method plot shows the relationship between the number of clusters and the dissimilarity measure.

Clustering based on the pattern of oral pain and its significantly associated/correlated variables were performed on 283 participants with complete data of all variables. Six was determined by the elbow method as the most appropriate number of clusters (Fig 4).

The final variable prototypes of each cluster are shown in Table 2. Detailed descriptive statistics (mean, standard deviation, 95% confidence interval, median and interquartile range) of each cluster is shown in S2 Table.

**Table 2:** Comparisons of variable prototypes between clusters.

Cluster	A	B	C	D	E	F
Number of participants	47	48	75	41	40	32
Oral pain (0 = never, 1 = hardly ever, 2 = occasionally to fairly often, 3 = very often)	0.15	0.27	0.36	0.49	0.78	2.19
Diastolic blood pressure (mmHg)	76.8	78.4	75.8	63.1	76.8	69.7
Red blood cell count (1000 cells/uL)	4.9	4.8	4.8	4	4.3	4.6
Haemoglobin (g/dL)	15.1	14.5	14.7	11.7	12.8	13.4
Haematocrit (%)	44.9	43.3	43.5	35.5	38.3	40.5
Depression (0-4 = none to minimal, 5-9 = mild, 10-14 = moderate, 15-19 = moderately severe, 20-27 = severe)	1.7	5.7	2.1	4	4.4	6.1
Excessive daytime sleepiness (0 = never, 1 = rarely, 2 = sometimes, 3 = often, 4 = almost always)	0.9	3.5	1.1	2.3	1.7	2.7
Having been told to take daily low-dose aspirin	No	Yes	Yes	Yes	No	Yes

**Fig 5:**
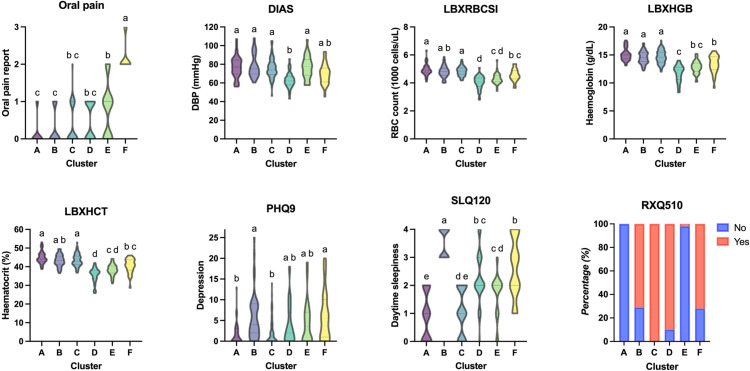
Visualisation and statistical comparisons of variables across clusters. At least one cluster did not pass the normality test for all continuous variables: diastolic blood pressure (DIAS), red blood cell count (LBXRBCSI), haemoglobin level (LBXHGB) and haematocrit (LBXHCT); hence, the Kruskal-Wallis test, followed by Dunn’s multiple comparisons test was performed on both continuous and ordinal variables, including oral pain, depression (PHQ9) and excessive daytime sleepiness (SLQ120). Different lowercase letters above cluster plots indicate significant differences between clusters (*p* < 0.05). The variable ‘having been told to take daily low-dose aspirin’ (RXQ510) is illustrated as a 100% stacked column graph; the Pearson’s chi-squared test demonstrated a significant association between this variable and cluster allocation (*p* < 0.05).

Cluster A represents a healthy group without significant clinical symptoms or extreme deviations in the haematologic variables. In contrast, Cluster F exhibits multimorbidity characteristics, reporting the highest degree of oral pain among clusters. Mild depression was also observed in Cluster F, with significantly higher levels than in Clusters A and C. In addition, Cluster F experienced a moderate degree of EDS, which was significantly higher than in Clusters A, C and E. The levels of DBP, RBC count, haemoglobin and haematocrit in Cluster F were moderate, compared to other clusters. Approximately 72% of Cluster F had been told to take daily low-dose aspirin. Though Cluster B also showed mild depression and the highest degree of EDS, they never or hardly every reported oral pain. A cluster reporting high oral pain without any comorbid symptoms was not observed (Fig 5). Of the 283 participants included in the cluster analysis, all six participants who reported very often oral pain also had at least one clinical comorbidity. Only two out of 35 participants reported occasionally to fairly often oral pain without comorbidities (S3 Table).

## Discussion

This study utilised a data mining approach to identify associated/correlated variables of non-odontogenic oral pain and their patterns in edentulous older people. Due to the mixed characteristics of the data, unsupervised machine learning with k-prototypes was used to explore subgroups of patterns within the population. In line with previous studies [24, 25], our research further showed that non-odontogenic oral pain was associated with certain systemic comorbidities in older people, as indicated by Cluster F. Notably, a cluster presenting solely with oral pain was not observed, suggesting that oral pain in the older people is not an isolated condition but often accompanied by other health problems.

### Depression

A large body of evidence shows a strong association between depression and orofacial pain, including TMD pain, burning mouth syndrome (BMS) [26,27] and neuropathic pain [28,29], as reiterated in this study. The relationship between mental illness, including depression, and pain is suggested to be bidirectional [30]. In our study, Cluster F reported both mild depression and frequent oral pain, whereas Cluster B, which also showed mild depression, only reported minimal oral pain. Thus, our findings fit into the paradigm that pain might serve as an aetiologic factor of depression [31]. Depression is a multifactorial disorder, and pain can induce depression through several mechanisms [32,33]. Pain has been shown to damage the dopaminergic system, which is crucial for mood regulation, leading to the development of depression [33]. In addition, orofacial pain patients often experience impaired quality of life [1], and stressful life events are associated with the onset of depression [34]. This, however, does not invalidate the bidirectional nature of pain and depression but suggests that depression might be more of a risk factor rather than a direct aetiologic factor of pain [35].

### Daytime sleepiness, snoring and apnoea

Studies on orofacial pain and sleep in the older people are rare, with most studies focusing on BMS [26,27]. Our study found that non-odontogenic oral pain is associated with EDS, which is consistent with a recent systematic review on BMS [36]. Studies on TMD pain patients also indicate that those experiencing pain have poorer sleep quality, compared to healthy individuals [37–39]. In our study, Cluster F reported moderate levels of oral pain and EDS. In addition, EDS was observed in Clusters B and D, which did not report significant pain, suggesting that oral pain might precede EDS. This is further supported by previous research showing that persistent pain is a predictor of EDS [40, 41]. Conversely, the coexistence of EDS and pain might imply a common factor, such as sleep disruption associated with sleep apnoea [42].

Unlike other studies we did not find a significant association between sleep apnoea and oral pain. This discrepancy could be due to the self-reported nature of the data we used, as self-reported EDS tends to be more reliable than self-reported snoring and sleep apnoea, especially in mild cases. Another explanation is that the association between sleep apnoea and pain is mediated via nocturnal hypoxia—arterial desaturation—independent of sleep fragmentation [43]. Thus, the NHANES questionnaire, which asked about the frequency of gasping per week, may not accurately reflect this association.

### Haematological variables

Our study showed that there was a negative relationship between levels of oral pain and several haematological features, including DBP, RBC count, haemoglobin levels and haematocrit. Such relationship has not been extensively documented, compared to psychosocial and sleep-related factors. Cluster analysis revealed that the high oral pain Cluster F had slightly but significantly lower levels of these haematological features, except for the DBP, compared to the healthy Cluster A, although these values remained close the normal range [44,45]. A previous study reports that TMD patients with lower mean arterial blood pressure have higher pain levels, though not statistically significant, compared to those with higher blood pressure [46]. However, given the multifactorial nature of orofacial pain, it not inconceivable that such small changes might contribute to pain initiation or maintenance when combined with other factors. Reduced RBC count, haemoglobin and haematocrit can impair vascular function [47] and oxygen saturation [48–50]; reduced oxygen saturation can lead to ischemic muscle pain, similar to what is observed in TMDs [51]. Another explanation is that low haematological features may reflect the effects of aspirin use as a secondary prevention of cerebrovascular accidents (CVA). Use of aspirin has been reported to lower RBC, haemoglobin and haematocrit [52]. Thus, the observed association between oral pain and haematological parameters might be related to a common aetiologic factor, such as past CVA.

In the present study, oral pain was found significantly associated with ‘having been told to take daily low-dose aspirin’, a characteristic also observed in the high oral pain Cluster F. However, no direct evidence linking low-dose aspirin use to oral pain has been reported. Previous studies have shown that past CVA can cause dysfunction in certain brain areas, leading to the development of chronic oral pain such as BMS [53,54]. Similarly, myocardial infarction can manifest as oral pain, with a reported prevalence of up to 38% [55]. Thus, the observed association between oral pain and being advised to take daily low-dose aspirin is likely due to the underlying medical conditions, such as CVA and myocardial infarction, in which oral pain is a secondary symptom and preventive use of aspirin is prescribed.

### Limitations

As a national survey, the granularity of the NHANES dataset is limited, preventing precise identification of the origin of the oral pain reported by participants. Given that all included participants were completely edentulous, it can be speculated that the pain was at least non-odontogenic. However, non-odontogenic pain encompasses a variety of subtypes, and the exact subtypes of the pain cannot be determined. In addition, denture use, a potential aetiologic factor of oral pain in our edentulous cohort, was not recorded, limiting our ability to assess its contribution. We were also unable to distinguish whether the pain was acute or chronic, which is crucial when discussing associated factors of pain. Cognitive status plays a significant role in pain perception and was not accounted for in the NHANES dataset. Nonetheless, it is reasonable to presume that participants who reported frequent oral pain were more likely to experience chronic pain, which is often linked to comorbidities such as depression, which is in line with our findings regarding Cluster F. Due to the eligibility criteria in the present study, our findings may not be generalisable to individuals aged 80 and older. Additionally, NHANES data is cross-sectional, limiting our ability to infer causality. Future research should refine methodologies to clarify pending unclear associations, particularly those related to haematological features.

## Conclusions

This study identified a cluster of edentulous older people living with oral pain in association with depression, daytime sleepiness and having been told to take daily low-dose aspirin. In addition, the cluster had lower RBC count, haemoglobin levels and haematocrit. The relationship between oral pain and haematological variables remains unclear and warrants further investigation in future studies.

## Supporting information

S1 Table Variables, assessment and possible responses from the NHANES 2017 – March 2020 pre-pandemic dataset used in the current study. (DOCX)

S2 Table Descriptive statistics for each cluster. (DOCX)

S3 Table A contingency table shows the frequency distribution of the clustered participants according to their oral pain reports and whether they have any clinical comorbidity. (DOCX)

S1 File Dataset of the included participants and cluster assignment. (XLSX)
