# Supplementary material for: Oral pain and comorbidities in an edentulous older population: A k-prototypes cluster analysis
Source: PLoS One. 2025 Mar 13;20(3):e0319819. doi: 10.1371/journal.pone.0319819 (PMC11906073; doi:10.1371/journal.pone.0319819)
Supplement: S1 Table — (DOCX) [file pone.0319819.s001.docx]

**S1 Table. Variables, assessment and possible responses from the NHANES 2017 – March 2020 pre-pandemic dataset used in the current study**

| **Variable** | **Assessment** | **Possible responses** |
| --- | --- | --- |
| Demographic data | | |
| RIAGENDR | Gender of the participant | Male, female |
| RIAGEYR | Age in years of the participant at the time of screening. Individuals 80 and over are topcoded at 80 years of age. | 0-79, 80 (80 years of age and over) |
| DMDEDUC2 | What is the highest grade or level of school you have completed or the highest degree you have received? | Less than 9^th^ grade, 9-11^th^ grade (including 12^th^ grade with no diploma), high school graduate/GED or equivalent, some college or AA degree, college graduate or above, refused, don’t know |
| INDFMPIR | A ratio of family income to poverty. | 0-4.98, 5.00 (value greater than or equal to 5.00) |
| Examination data | | |
| BPXOSY1-3 | After resting quietly in a seated position for 5 minutes; three consecutive blood pressure and pulse rate measurements were taken 60 seconds apart using a digital upper-arm electronic measurement device, Omron HEM–907XL. Measurements were taken in the right arm unless specific conditions prohibited the use of the right arm, or if participants reported any reason that the measurements should not be taken in the right arm. | 52-225 |
| BPXODI1-3 |  | 28-151 |
| BPXOPLS1-3 |  | 34-147 |
| BMXBMI | Body mass index (kg/m^2^) | 11.9-92.3 |
| OHX01TC -OHX32TC | Tooth count | Primary tooth present, permanent tooth present, dental implant, teeth not present, permanent dental root fragment present, could not assess |
| Laboratory data | | |
| CBC | The complete blood count (CBC) with 5-part differential: counts red blood cells (RBCs), white blood cells (WBCs), and platelets; measures hemoglobin; estimates the RBC’s volume; and sorts the WBCs into subtypes.  The Beckman Coulter DxH 800 instrument, in the NHANES mobile examination center (MEC), was used to measure the CBC on blood specimens and provide a distribution of blood cells for all participants. The methods used to derive CBC parameters are based on the Beckman Coulter methodology of counting and sizing, in combination with an automatic diluting and mixing device for sample processing, and a single beam photometer for hemoglobinometry. The WBC differential uses VCS (volume, conductivity and scatter) technology. |  |
| LBXWBCSI | White blood cell count (1000 cells/uL) | 1.9-74.2, 400 (400 and over) |
| LBXLYPCT | Lymphocyte percent (%) | 3.1-89.7 |
| LBXMOPCT | Monocyte percent (%) | 0.7-57.2 |
| LBXNEPCT | Segmented neutrophils percent (%) | 8.4-92.8 |
| LBXEOPCT | Eosinophils percent (%) | 0-29.1 |
| LBXBAPCT | Basophils percent (%) | 0.1-4.8 |
| LBDLYMNO | Lymphocyte number (1000 cells/uL) | 0.2-358.8 |
| LBDMONO | Monocyte number (1000 cells/uL) | 0.1-6.7 |
| LBDNENO | Segmented neutrophils number (1000 cell/uL) | 0.4-35.2 |
| LBDEONO | Eosinophils number (1000 cells/uL) | 0-3.8 |
| LBDBANO | Basophils number (1000 cells/uL) | 0-0.5 |
| LBXRBCSI | Red blood cell count (million cells/uL) | 2.32-7.97 |
| LBXHGB | Hemoglobin (g/dL) | 5.4-19.9 |
| LBXHCT | Hematocrit (%) | 21.1-58.8 |
| LBXMCVSI | Mean cell volume (fL) | 35.4-114.6 |
| LBXMC | Mean cell hemoglobin concentration (g/dL) | 25.2-38.4 |
| LBXMCHSI | Mean cell hemoglobin (pg) | 10.2-39.8 |
| LBXRDW | Red cell distribution width (%) | 11.3-36.5 |
| LBXPLTSI | Platelet count (1000 cells/uL) | 8-1021 |
| LBXMPSI | Mean platelet volume (fL) | 5.4-13 |
| LBXNRBC | Nucleated red blood cells | 0-3 |
| LBXFER | The method for the measurement of Ferritin on the Roche Cobas® e601 is a sandwich principle with a total duration time of 18 minutes. The 1^st^incubation uses 10 μL of sample, a ferritin-specific antibody and a labeled ferritin-specific antibody to form a sandwich complex. The 2^nd^ incubation occurs after the addition of microparticles that cause the complex to bind to the solid phase. The reaction mixture is aspirated into the measuring cell where the microparticles are magnetically captured onto the surface of the electrode. Unbound substances are then removed. Application of a voltage to the electrode then induces chemiluminescent emission which is measured by a photomultiplier. Results are determined via a calibration curve.  Ferritin (ng/mL) | 1.04-5190 |
| LBXGH | In this assay, the stable (SA1c) and labile (LA1c) A1c forms can be individually resolved on the chromatogram without manual pretreatment, allowing accurate measurement of the stable form of HbA1c. The analyzer dilutes the whole blood specimen with a hemolysis solution, and then injects a small volume of the treated specimen onto the HPLC analytical column. Separation is achieved by utilizing differences in ionic interactions between the cation exchange group on the column resin surface and the hemoglobin components. The hemoglobin fractions (A1c, A1b, F, LA1c, SA1c, A0 and H-Var) are subsequently removed from the column material by step-wise elution using elution buffers each with a different salt concentration. The separated hemoglobin components pass through the photometer flow cell where the analyzer measures changes in absorbance at 415 nm. The analyzer integrates and reduces the raw data, and then calculates the relative percentages of each hemoglobin fraction. Analysis requires three minutes. If a specimen showed a deterioration peak, hemoglobin variant, or a LA1c results ≥ 5% and/or LA1c results > half SA1c during the regular test, it would be retested by a second method, ultra 2 HPLC. In the 2017March 2020 pre-pandemic sample, only 3.9% of the blood specimens required to be retested by the ultra 2 HPLC method. A lab instrumentation change was made for this secondary method during the data collection period. This instrument change did not significantly affect the resulted glycohemoglobin values; the mean relative error between results from the two instruments was 0.5% (ranged from -4.0% to 5.2%).  Glycohemoglobin (%) | 2.8-16.2 |
| LBXGLU | In this enzymatic method glucose is converted to glucose-6-phosphate (G-6-P) by hexokinase in the presence of ATP, a phosphate donor. Glucose-6-phosphate dehydrogenase then converts the G-6-P to gluconate-6-P in the presence of NADP+. As the NADP+ is reduced to NADPH during this reaction, the resulting increase in absorbance at 340 nm (secondary wavelength = 700 nm) is measured. This is an endpoint reaction that is specific for glucose.  Fasting Glucose (mg/dL) | 47-524 |
| Questionnaire data | | |
| OHQ620 | How often during the last year have you had painful aching anywhere in your mouth? | Very often, fairly often, occasionally, hardly ever, never, refused, don’t know |
| BPQ020 | {Have you/Has SP} ever been told by a doctor or other health professional that {you/s/he} had hypertension, also called high blood pressure? | Yes, no, refused, don’t know |
| BPQ050A | {Are you/Is SP} now taking prescribed medicine? | Yes, no, refused, don’t know |
| BPQ080 | {Have you/Has SP} ever been told by a doctor or other health professional that {your/his/her} blood cholesterol level was high? | Yes, no, refused, don’t know |
| BPQ100D | (Are you/Is SP) now following this advice to take prescribed medicine? | Yes, no, refused, don’t know |
| CDQ001 | {Have you/Has SP} ever had any pain or discomfort in {your/her/his} chest? | Yes, no, refused, don’t know |
| CDQ010 | {Have you/Has SP} had shortness of breath either when hurrying on the level or walking up a slight hill? | Yes, no, refused, don’t know |
| DIQ010 | {Other than during pregnancy, {have you/has SP}/{Have you/Has SP}} ever been told by a doctor or health professional that {you have/{he/she/SP} has} diabetes or sugar diabetes? | Yes, no, borderline, refused, don’t know |
| DIQ050 | {Is SP/Are you} now taking insulin | Yes, no, refused, don’t know |
| DPQ010-090 | A nine-item depression screening instrument (variable prefix is DPQ), also called the Patient Health Questionnaire (Kroenke and Spitzer, 2002; Kroenke et al., 2001) was administered to determine the frequency of depression symptoms over the past 2 weeks. For each symptom question, points ranging from 0 to 3, are associated with the response categories "not at all," "several days," "more than half the days," and "nearly every day.” | Not at all, several days, more than half the days, nearly every day, refused, don’t know |
| RXQ510 | Doctors and other health care providers sometimes recommend that {you take/SP takes) a low-dose aspirin each day to prevent heart attacks, strokes, or cancer. {Have you/Has SP} ever been told to do this? | Yes, no, refused, don’t know |
| SLQ030 | In the past 12 months, how often did {you/SP} snore while {you were/s/he was} sleeping? | Never, rarely—1-2 nights a week, occasionally—3-4 nights a week, frequently—5 or more nights a week, refused, don’t know |
| SLQ040 | In the past 12 months, how often did {you/SP} snort, gasp, or stop breathing while {you were/s/he was} asleep? | Never, rarely—1-2 nights a week, occasionally—3-4 nights a week, frequently—5 or more nights a week, refused, don’t know |
| SLQ120 | In the past month, how often did {you/SP} feel excessively or overly sleepy during the day? | Never, rarely—1 time a month, sometimes—2-4 times a month, often—5-15 times a month, almost always—16-30 times a month, refused, don’t know |
