# Supplementary material for: Oral pain and comorbidities in an edentulous older population: A k-prototypes cluster analysis
Source: PLoS One. 2025 Mar 13;20(3):e0319819. doi: 10.1371/journal.pone.0319819 (PMC11906073; doi:10.1371/journal.pone.0319819)
Supplement: S2 Table — (DOCX) [file pone.0319819.s002.docx]

**S2 Table. Descriptive statistics for each cluster.**

| **Cluster** | | **A** | **B** | **C** | **D** | **E** | **F** |
| --- | --- | --- | --- | --- | --- | --- | --- |
| Number of participants | | 47 | 48 | 75 | 41 | 40 | 32 |
| Oral pain (0=never, 1=hardly ever, 2=occasionally to fairly often, 3=very often) | Mean | 0.15 | 0.27 | 0.36 | 0.49 | 0.78 | 2.19 |
|  | SD | 0.36 | 0.45 | 0.54 | 0.51 | 0.73 | 0.4 |
|  | Lower 95% CI | 0.04 | 0.14 | 0.24 | 0.33 | 0.54 | 2.04 |
|  | Upper 95% CI | 0.26 | 0.4 | 0.48 | 0.65 | 1.01 | 2.33 |
|  | Median | 0 | 0 | 0 | 0 | 1 | 2 |
|  | IQR | 0 | 1 | 1 | 1 | 1 | 0 |
| Diastolic blood pressure (mmHg) | Mean | 76.8 | 78.4 | 75.8 | 63.1 | 76.8 | 69.7 |
|  | SD | 11.7 | 12.4 | 10.8 | 8.42 | 11.8 | 11.0 |
|  | Lower 95% CI | 73.3 | 74.8 | 73.3 | 60.4 | 73.0 | 65.8 |
|  | Upper 95% CI | 80.2 | 82.0 | 78.2 | 65.7 | 80.6 | 73.7 |
|  | Median | 77.0 | 75.0 | 74.0 | 62.3 | 77.7 | 70.5 |
|  | IQR | 14.5 | 19.3 | 12.5 | 7.7 | 15.6 | 14.5 |
| Red blood cell count (1000 cells/uL) | Mean | 4.9 | 4.8 | 4.8 | 4 | 4.3 | 4.6 |
|  | SD | 0.4 | 0.4 | 0.3 | 0.5 | 0.4 | 0.4 |
|  | Lower 95% CI | 4.8 | 4.7 | 4.8 | 3.9 | 4.2 | 4.4 |
|  | Upper 95% CI | 5.1 | 4.9 | 4.9 | 4.2 | 4.4 | 4.7 |
|  | Median | 4.9 | 4.8 | 4.9 | 4.1 | 4.3 | 4.5 |
|  | IQR | 0.4 | 0.5 | 0.4 | 0.6 | 0.4 | 0.5 |
| Haemoglobin (g/dL) | Mean | 15.1 | 14.5 | 14.7 | 11.7 | 12.8 | 13.4 |
|  | SD | 1.1 | 1.1 | 1.2 | 1.5 | 1.1 | 1.6 |
|  | Lower 95% CI | 14.8 | 14.2 | 14.4 | 11.2 | 12.4 | 12.8 |
|  | Upper 95% CI | 15.4 | 14.9 | 14.9 | 12.2 | 13.2 | 14.0 |
|  | Median | 15 | 14.5 | 14.6 | 12.3 | 12.9 | 13.6 |
|  | IQR | 1.4 | 1.6 | 1.7 | 2.2 | 1.3 | 2.2 |
| Haematocrit (%) | Mean | 44.9 | 43.3 | 43.5 | 35.5 | 38.3 | 40.5 |
|  | SD | 3.3 | 3.2 | 3.3 | 4 | 3 | 3.9 |
|  | Lower 95% CI | 43.9 | 42.4 | 42.8 | 34.2 | 37.3 | 39.0 |
|  | Upper 95% CI | 45.8 | 44.2 | 44.3 | 36.7 | 39.2 | 41.9 |
|  | Median | 44.1 | 43.4 | 42.9 | 36.6 | 38.2 | 41.2 |
|  | IQR | 3.8 | 4.3 | 4.3 | 4.2 | 3.6 | 5.3 |
| Depression (0-4=none to minimal, 5-9=mild, 10-14=moderate, 15-19=moderately severe, 20-27=severe) | Mean | 1.7 | 5.7 | 2.1 | 4 | 4.4 | 6.1 |
|  | SD | 2.5 | 5.5 | 3 | 4.7 | 5 | 5.8 |
|  | Lower 95% CI | 1 | 4.1 | 1.4 | 2.5 | 2.8 | 4.0 |
|  | Upper 95% CI | 2.4 | 7.3 | 2.8 | 5.4 | 6 | 8.2 |
|  | Median | 1 | 4 | 1 | 2 | 3.5 | 5.5 |
|  | IQR | 3 | 7 | 3 | 6 | 7 | 9 |
| Excessive daytime sleepiness (0=never, 1=rarely, 2=sometimes, 3=often, 4=almost always) | Mean | 0.9 | 3.5 | 1.1 | 2.3 | 1.7 | 2.7 |
|  | SD | 0.8 | 0.5 | 0.8 | 0.9 | 0.8 | 1.1 |
|  | Lower 95% CI | 0.7 | 3.4 | 0.9 | 2 | 1.4 | 2.3 |
|  | Upper 95% CI | 1.1 | 3.7 | 1.2 | 2.6 | 1.9 | 3 |
|  | Median | 1 | 4 | 1 | 2 | 2 | 3 |
|  | IQR | 2 | 1 | 2 | 1 | 1 | 2 |
| Having been told to take daily low-dose aspirin | Count (Yes) | 0 | 34 | 75 | 37 | 1 | 23 |
|  | % (Yes) | 0% | 70.8% | 100% | 90.2% | 2.5% | 71.9% |

SD, standard deviation; CI, confidence interval.
