# Supplementary material for: Oral pain and comorbidities in an edentulous older population: A k-prototypes cluster analysis
Source: PLoS One. 2025 Mar 13;20(3):e0319819. doi: 10.1371/journal.pone.0319819 (PMC11906073; doi:10.1371/journal.pone.0319819)
Supplement: S3 Table — (DOCX) [file pone.0319819.s003.docx]

**S3 Table. A contingency table shows the frequency distribution of the clustered participants according to their oral pain reports and whether they have any clinical comorbidity.**

|  | Oral pain report | | | |
| --- | --- | --- | --- | --- |
| Clinical comorbidity | Never (n=162) | Hardly ever (n=80) | Occasionally to fairly often (n=35) | Very often (n=6) |
| No | 28 | 9 | 2 | 0 |
| Yes | 134 | 71 | 33 | 6 |

Clinical comorbidity is defined as having at least one of the following: mild or greater depression (PHQ9>4), excessive daytime sleepiness felt sometimes or more frequently (SLQ120>1), or ‘having been told to take daily low-dose aspirin’.
